# Bis-anthracycline WP760 abrogates melanoma cell growth by transcription inhibition, p53 activation and IGF1R downregulation

**DOI:** 10.1007/s10637-017-0465-9

**Published:** 2017-04-17

**Authors:** Magdalena Olbryt, Aleksandra Rusin, Izabela Fokt, Anna Habryka, Patrycja Tudrej, Sebastian Student, Aleksander Sochanik, Rafał Zieliński, Waldemar Priebe

**Affiliations:** 1 Center for Translational Research and Molecular Biology of Cancer, Maria Skłodowska-Curie Memorial Cancer Center and Institute of Oncology, Gliwice Branch, Wybrzeże Armii Krajowej 15, 44-101, Gliwice, Poland; 2 Department of Experimental Therapeutics, University of Texas MD Anderson Cancer Center, Houston, TX USA; 3 Faculty of Automatic Control, Electronics and Computer Science, Silesian University of Technology, Gliwice, Poland

**Keywords:** WP760, Anthracyclines, p53, Transcriptional inhibitor, Melanoma

## Abstract

Anthracycline chemotherapeutics, e.g. doxorubicin and daunorubicin, are active against a broad spectrum of cancers. Their cytotoxicity is mainly attributed to DNA intercalation, interference with topoisomerase activity, and induction of double-stranded DNA breaks. Since modification of anthracyclines can profoundly affect their pharmacological properties we attempted to elucidate the mechanism of action, and identify possible molecular targets, of bis-anthracycline WP760 which previously demonstrated anti-melanoma activity at low nanomolar concentrations. We studied the effect of WP760 on several human melanoma cell lines derived from tumors in various development stages and having different genetic backgrounds. WP760 inhibited cell proliferation (IC_50_ = 1–99 nM), impaired clonogenic cell survival (100 nM), and inhibited spheroid growth (≥300 nM). WP760 did not induce double-stranded DNA breaks but strongly inhibited global transcription. Moreover, WP760 caused nucleolar stress and led to activation of the p53 pathway. PCR array analysis showed that WP760 suppressed transcription of ten genes (*ABCC1, MTOR, IGF1R, EGFR, GRB2, PRKCA, PRKCE, HDAC4, TXNRD1, AKT1*) associated with, inter alia, cytoprotective mechanisms initiated in cancer cells during chemotherapy. Furthermore, WP760 downregulated IGF1R and upregulated *PLK2* expression in most of the tested melanoma cell lines. These results suggest that WP760 exerts anti-melanoma activity by targeting global transcription and activation of the p53 pathway and could become suitable as an effective therapeutic agent.

**Electronic supplementary material:**

The online version of this article (doi:10.1007/s10637-017-0465-9) contains supplementary material, which is available to authorized users.

## Introduction

Anthracyclines and their derivatives have been widely used for the last half century to treat several types of neoplasms. Cytotoxicity and anticancer activity of anthracyclines have been attributed to modes of action including interaction with DNA and type II topoisomerases-mediated histone removal from chromatin, inhibition of DNA and RNA synthesis, and generation of reactive oxygen species [1, 2]. The prevailing mechanism responsible for action of anthracycline derivatives may differ depending on the nature of chemical substitutions.

Anthracyclines bind to topoisomerase IIa–DNA complex and induce ternary complex formation that is associated with inhibition of topoisomerase II. This interferes with DNA ligation, leading to the formation of double-stranded breaks [3]. This mode of action, however, is a double edged-sword as topoisomerase inhibition and/or poisoning in cardiomyocytes leads to dose-limiting cardiotoxicity; cumulative doses of anthracyclines result in cytotoxicity that may manifest as cardiomyopathy leading to congestive heart failure [2].

Melanomas are either resistant to chemotherapy or develop resistance during treatment, and residual chemoresistant cells are highly metastatic [4]. The main strategies currently used for treating advanced melanoma include targeted therapies (e.g. BRAF inhibitors, such as vemurafenib) and immunotherapy (e.g. anti-CTLA-4 antibodies, such as ipilimumab) [5]. Owing to inherent or acquired resistance to treatment, metastatic melanoma remains largely incurable. Therefore, novel melanoma drugs are urgently needed.

Previously, using a “lego block”-type assembly of bis-anthracyclines, we synthesized a mini library of third-generation analogs varying in terms of lipophilicity, DNA affinity, and biological activity. Screening experiments led to the identification of WP760, a bis-anthracycline with high cytotoxic potential and remarkable selectivity towards melanoma cells [6, 7]. This study attempted to elucidate the molecular mechanism of WP760 action and to further determine biochemical properties of this compound. The antiproliferative activity of WP760 was investigated in a broad panel of human melanoma cell lines cultured under various conditions including hypoxic microenvironment and 3D spheroids. Anthracyclines and their derivatives have been widely used for treating several types of neoplasms in the last half century. Cytotoxicity and anticancer activity of anthracyclines have been attributed to their various modes of action, including interaction with DNA and type II topoisomerases-mediated histone removal from chromatin, which affect DNA damage response, inhibition of DNA and RNA synthesis, and generation of reactive oxygen species that damage macromolecules and cell membranes [1, 2]. However, the prevailing mechanism responsible for action of different anthracycline derivatives may differ depending on the nature of chemical substitutions.

## Materials and methods

### Cells, reagents, hypoxic conditions

Melanoma cell lines were cultured as recommended by repositories and routinely tested for mycoplasma contamination and authenticated by SNP fingerprinting (LGC Standards, Poland). Media were supplemented with FBS and Antibiotic Solution (Sigma-Aldrich). For details see Supplementary material, Table S1. WP760 and doxorubicin were dissolved in DMSO (150 μM and 10 mM stocks, respectively) and stored at 4 °C. Spheroids were formed by seeding 1 × 10^4^ cells/200 μL medium in 96-well plates pre-coated with 1% noble agar. After formation of compact spheroids, cultures were continued in ultra-low-attachment 96-well plates. Subsequently, WP760 was added on 4th and 7th day after seeding. Medium (100 μL) was replaced 3 times/week. For culture under hypoxic conditions, cells were kept in an oxygen-controlled incubator (1% O_2_).

### Cytotoxicity assay

WP760 (and doxorubicin) cytotoxicity was assessed using MTS assay (Promega, G3581). Cells were seeded in 96-well plates, cultured for 24 h and treated with WP760. MTS assay was performed after 72-h incubation. Absorbance was read using a Biotek reader. Half-maximal inhibitory concentration (IC_50_) was calculated by sigmoidal curve fitting using non-linear least squares regression analysis in the R Stats Package developed by R Core Team [8] or GraphPad Prism. Linear approximation was used to calculate the 95% confidence intervals of log (IC_50_) estimates. WP760 cytotoxicity in spheroid cultures was evaluated by measuring spheroid diameter every 2–5 days using Olympus IX81 microscope (Olympus Europa GmbH, Hamburg, Germany).

### In vitro clonogenic assay

Cells were incubated for 4 h in the presence of WP760 (100 nM) or 0.067% DMSO (control) at 37 °C, trypsinized, reseeded at 1.5–2.0 × 10^3^ cells/3-cm polystyrene plates, and incubated until the formation of colonies (11–28 days, depending on cell line). The medium was changed once a week. Colonies were fixed with methanol/formalin 1:1 (*v*/v), stained with 0.5% crystal violet for 15 min at 37 °C, and counted using G:Box Imaging System and GeneTools software (Syngene) or Image J (NIH).

### Cell cycle analysis

Cells were incubated for 24 h with 100 nM WP760 (where necessary the treatment was preceded by 24-h incubation with BSA-free medium to partially synchronize cells). Subsequently, cells were harvested, rinsed with PBS, fixed using 70% EtOH, stained with propidium iodide (Sigma-Aldrich) and analyzed using FACSCanto flow cytometer (Becton Dickinson) equipped with 488 nm argon laser. Percentage of cells at each phase of the cell cycle was estimated using FACSDiva software.

### Apoptosis analysis

Apoptosis was analyzed using TUNEL staining (Roche, 11684795910). Cells seeded in 8-well glass chambers were incubated with WP760 (24–48 h) and treated with TUNEL reaction mixture (slight modification). Briefly, 200 μL of TUNEL reaction mixture was added, cells were covered with coverslips and incubated (60 min/37 °C/dark) in a humidified atmosphere. Subsequently, cells were stained with DAPI (1 μg/mL) and analyzed using confocal microscopy (Zeiss LSM750). Apoptotic index was determined by counting at least 1000 neoplastic nuclei subdivided in 6–10 randomly chosen fields at 200× magnification. Apoptotic cells were identified by TUNEL staining status together with characteristic morphological features (cell shrinkage, membrane blebbing, and chromatin condensation).

### RNA isolation

RNA isolation was performed using RNeasy Mini Kit (Qiagen). Additional homogenization step (QIAshredder), or homogenization by passing through a 21-gauge needle, and DNase I (Qiagen) digestion were also performed. Quality of isolated RNA was assessed using 2100 Bioanalyzer (Agilent Technologies). RNA was quantified by absorbance at 260 nm using a NanoDrop (Thermo Scientific).

### PCR array analysis

RT^2^ Profiler PCR Array (Qiagen, PAHS-507Z) was used for gene expression analysis. Cells at approx. 70% confluency were incubated with 100 nM WP760 or DMSO (control) for 24 h. Subsequently, cells were trypsinized, washed with PBS-, lysed with RLT buffer containing β-ME (Qiagen), and stored at −80 °C until RNA isolation. Total RNA (3 μg) was reverse-transcribed into cDNA (Qiagen, 330,404) and qPCR was performed using Qiagen reagents (330502) and CFX96 thermocycler (Bio-Rad).

RNA isolation was performed using RNeasy Mini Kit (Qiagen) following the manufacturer’s protocol. An additional homogenization step (QIAshredder) or homogenization by passing through a 21-gauge needle, and DNase I (Qiagen) digestion were also performed. The quality of isolated RNA was assessed using 2100 Bioanalyzer (Agilent Technologies). RNA was quantified by absorbance measurement at 260 nm using a NanoDrop spectrophotometer (Thermo Scientific).

### Quantitative reverse transcription polymerase chain reaction (qRT-PCR)

qRT-PCR was performed using GoTaq® 2-Step RT-qPCR System (Promega, A6010). Briefly, 1 μg of total RNA was used for cDNA synthesis (25 μL total volume) using a C1000 thermocycler (BioRad). cDNA was diluted 5-fold and 5-μL aliquots were used for Real-Time PCR performed on CFX96 Real-Time System (BioRad): 2 min at 95 °C, 40 cycles of 15 s at 95 °C and 1 min at 60 °C; final melting at 60–95 °C. Gene expression was calculated as previously described [9]. Reference genes used for normalization were: *B2M* and *RPLP0* for WM35, 451Lu, and WM1382 cell lines; *PLXNB2* and *RPLP0* for all other melanoma cell lines (for sequences see Supplementary material, Table S2).

### Western blotting

Cells were trypsinized, washed with PBS^−^, and lysed in IP or RIPAS buffer supplemented with protease inhibitor cocktail (Roche). The lysates were incubated for 15 min on ice, centrifuged, and frozen. Immunoblotting was performed using aliquots (15–30 μg) of the whole-cell extract. Proteins were separated on polyacrylamide gels (8 or 12%) and blotted onto PVDF (Immobilon-P Transfer Membrane; Millipore) or nitrocellulose (Amersham Protran Premium) membranes. Antibodies used for detection are listed in Supplementary material (Table S3). The chemiluminescent substrate signal (Thermo Scientific; 34079, 34095) was developed using the Curix60 processor (Agfa).

### Global RNA synthesis determination

Global RNA synthesis was evaluated using Click-it RNA imaging kit (Thermo Fisher Scientific, C10330). Cells were grown on glass coverslips for 24 h in complete culture medium containing WP760 (10 nM, 50 nM, and 100 nM), or 25 nM actinomycin D, (Sigma-Aldrich). Briefly, staining was performed as follows: ethynyl uridine (EU) was added to the culture medium 1 h before the end of incubation with the tested compounds. Cells were then rinsed, fixed in 3.7% formaldehyde, and washed with PBS followed by staining with 10 μM Alexa594-azide. Subsequently, cells were washed with PBST, counterstained with Hoechst 33342, and visualized by confocal microscopy (Zeiss LSM750). Signal intensity was evaluated using ImageJ software.

### Immunofluorescent detection of B23

Cells were grown in 8-well glass chamber slides (Nunc). After fixation with paraformaldehyde (3.7% in PBS), cells were permeabilized with 0.05% Triton-X100 in PBS, blocked with FBS, and incubated (overnight, 4 °C) with mouse anti-B23 antibody (Sigma-Aldrich, B0556-100UL) diluted in PBS (1:200), washed thrice with PBS and incubated with secondary anti-mouse antibody conjugated with Alexa Fluor 488 for 1 h (RT). The slides were imaged under a confocal microscope (Zeiss LSM750).

### DNA mobility shift assay

Linearized pEBFP plasmids (BamH1) were used to evaluate the binding of WP760 to dsDNA. Plasmid DNA isolation was carried out using a Flex Prep kit (Amersham-Pharmacia Biotech). For DNA mobility shift assays, 1 μL plasmid DNA (100 ng/μL) was mixed with 1 μL of WP760 (desired concentration in DMSO). Doxorubicin was used as a reference. Final volume of each reaction was adjusted to 10 μL with MilliQ water. The sample mixtures were incubated for 20 min at 37 °C and electrophoresed on 1% agarose gel (0.5× TBE buffer; 5.3 V/cm, 2 h). Gels were stained with ethidium bromide and imaged (Gel Imager, BTX-20.M, Syngen).

### Decatenation assay

Decatenation assay was performed using a Topo II Assay Kit (TopoGEN, Inc., SKU TG1001–1). Briefly, kinetoplast DNA (0.2 g) was incubated (37 °C/15 min) with 2 units of Top2a (TopoGEN Inc., SKU TG2000H-1) in 20 μL of reaction buffer containing 5% DMSO and WP760 or doxorubicin (reference). One unit of activity was defined as the amount of Top2 enzyme that decatenates 0.2 g of kinetoplast DNA under standard conditions. The reaction was stopped by adding 5 μL of loading dye. The samples were then electrophoresed using 1% agarose gel (TBE buffer containing 0.5 g/mL of ethidium bromide).

### Histone ɣH2AX detection

Phosphorylated form of histone H2AX was detected by flow cytometry in immunofluorescently stained samples. Briefly, cells were grown for 24 h in 3-cm plates in the presence or absence of WP760 or doxorubicin (reference). Cells were then trypsinized, fixed with paraformaldehyde (3.8% in PBS), and permeabilized with Triton X-100 (0.05%). Subsequently, cells were incubated with primary antibody (1:500) followed by secondary FITC-conjugated antibody (1:1000). Fluorescence signals were analyzed using BD FACSCanto flow cytometer. Median fluorescence signal was calculated using FACSDiva software.

### Nuclear localization of WP760

WM35 or A375 cells seeded on glass chamber slides were incubated with 1 μM WP760 for 2, 4, or 24 h and examined with Delta Vision OMX imaging system or Olympus FLV1000 confocal microscope. Series of optical sections were deconvolved and overlaid (maximum projection mode).

### Statistical analysis

Statistical evaluation of the results was performed using Student’s t-test or Fisher's test. Data are expressed as mean ± SD. *P*-values <0.05 (*) were considered statistically significant, whereas *P*-values <0.01 (**) were considered highly significant.

## Results

### WP760 inhibits proliferation of melanoma cells at low nanomolar concentrations

We evaluated the antiproliferative activity of WP760 in a panel of 12 human melanoma cell lines derived from tumors at different development stage and with different genetic backgrounds. After 72-h treatment with WP760, MTS assay showed a dose-dependent inhibition of cell proliferation with IC_50_ values ranging from 1.4 to 99.6 nM (median 7.5 nM, Table 1). Similar cytotoxicity was observed for cell lines resistant to BRAF inhibitors (median 5.1 nM). Slightly decreased cytotoxicity was observed for cells cultured under hypoxic conditions (1% O_2_); however, the median IC_50_ remained very low (26 nM) (Table 1).

**Table 1:** IC_50_ values of WP760 for melanoma and non-melanoma cell lines

Cell Line	WP760	WP760 under hypoxia	Doxorubicin
1205Lu	4.0	4.8	NT
451Lu	42.1	137.4	NT
A375	2.6	NT	52.5
A375 VR	4.2	NT	NT
Mel1617	2.2	NT	NT
Mel1617 VR	2.7	NT	NT
SB2	1.4	NT	36.3
SKMel-28	27.4	NT	827
SKMel-28 VR	99.6	NT	NT
WM1382	21.5	31.2	1013.1
WM1552C	7.5	9.9	NT
WM278	21.5	26.0	NT
WM3248	3.8	8.5	410.1
WM35	27.3	76.45	290.9
WM3928F	16.2	163.5	587.2
WM793B	4.5	15.6	NT
WM793B VR	6.1	NT	NT
A549 (lung cancer)	40.1	NT	NT
OV-PA-8 (ovarian cancer)	53.9	NT	NT

*NT* not tested, *VR* vemurafenib resistant

**Fig. 1:**
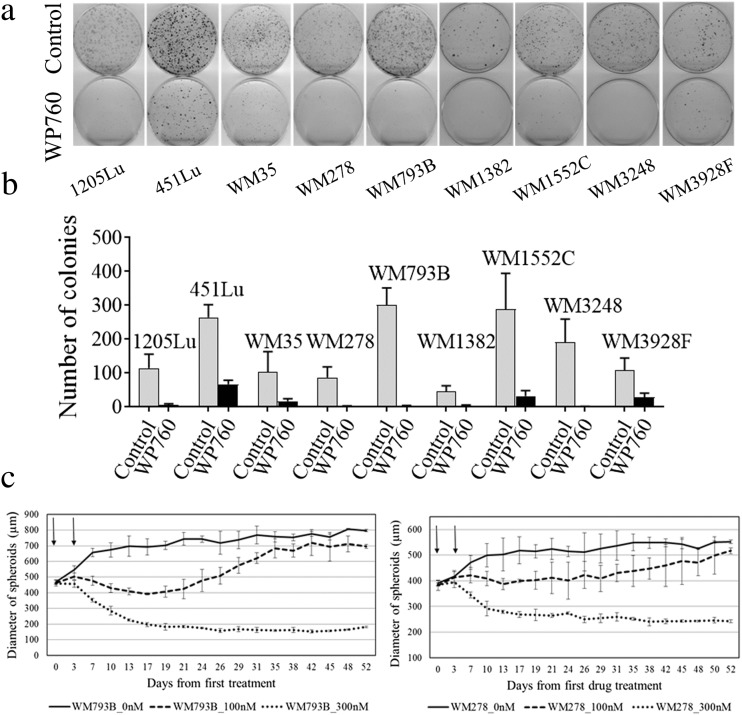
In vitro cytotoxicity assays of WP760. Clonogenicity assay on melanoma cells exposed to 100 nM WP760 or DMSO (control) for 4 h (**a**). Colonies were stained with crystal violet and counted using G:Box Imaging System (Syngen) or ImageJ program. Control and treated plates representative for both technical and experimental replicas are shown. The mean colony numbers ± SDs are shown for three experiments each containing 2–3 technical replicas (**b**). Influence of WP760 treatment on melanoma spheroids growth (**c**): WM793B (*left*) and WM278 (*right*) cell cultures treated with WP760 (100 nM or 300 nM) or DMSO on the 4th and 7th day of culturing (0 and 3rd day of treatment; arrows). Dots represent means of three independent experiments and the whiskers show standard deviation. For 100 nM WP760 the results are statistically significant (*P*-values < 0.05, Student’s t-test) in the time span from 7th to 31st day (WM793B) and from 7th to 42nd day (WM278). For 300 nM WP760, the results are statistically significant from the 3rd day on

WP760 affected clonogenic capacity of all tested melanoma cell lines. Treatment with 100 nM WP760 for 4 h either completely inhibited clonogenicity (WM793B, 1205Lu, WM3248, WM278, and WM1382 cell lines) or significantly reduced it (WM35, WM3928F, WM1552C, and 451Lu). Survival factor ranged from 1 to 25% (Fig. 1a, b).

WP760 (300 nM) significantly impaired spheroid growth in 3D cultures of WM793B and WM278 cell lines. Administration of two drug doses completely abrogated growth of spheroids. Moreover, remnant cell clusters did not re-grow during the 3-week culture that followed. In contrast, 100 nM WP760 only slightly inhibited the WM278 spheroid growth and destroyed outer cell layer in WM793B spheroids. Both cell lines started to re-grow approximately 3 weeks post-treatment, reaching size of control spheroids after 6–7 weeks (Fig. 1c). WP760 also inhibited non-melanoma cells (ovarian and lung) growth, but due to more potent anti-melanoma properties we focused our analysis on melanoma cell lines. In order to analyze biochemical and molecular effects of WP760 at doses causing >50% cell cytotoxicity, all experiments were performed using drug concentrations exceeding median IC_50_.

### Cell cycle and apoptosis

**Fig. 2:**
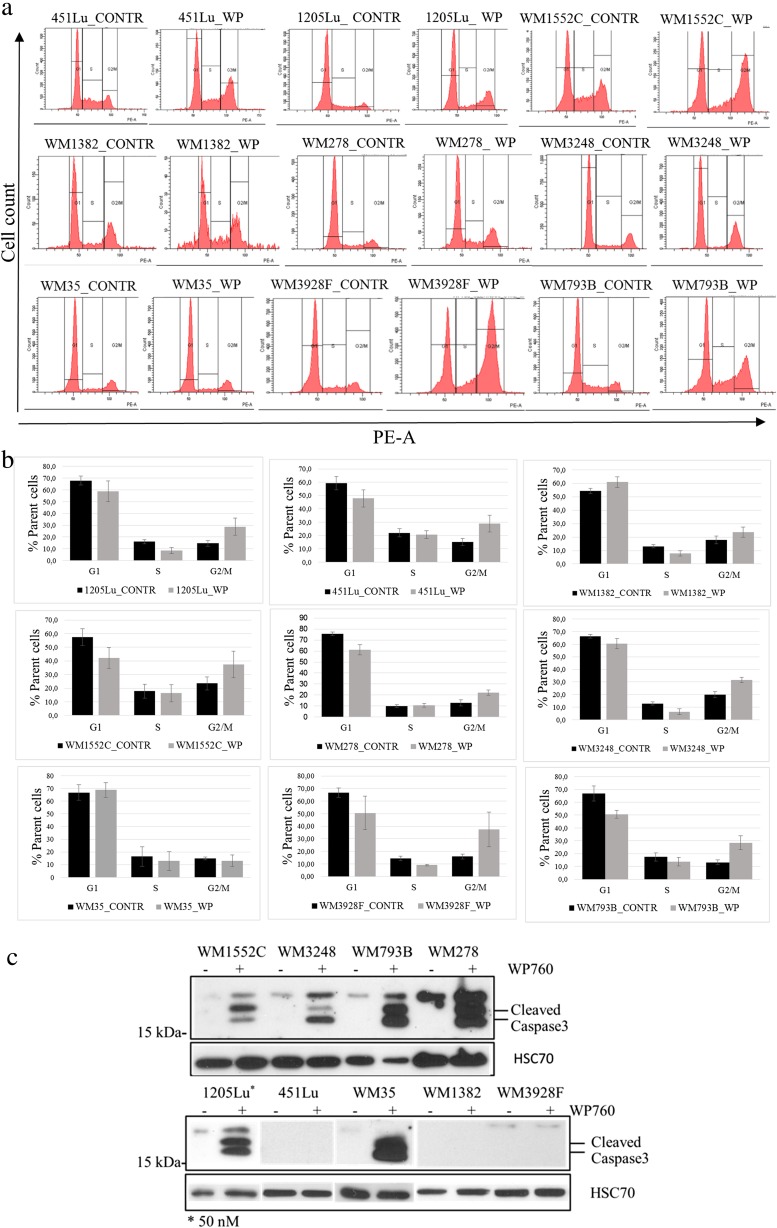
WP760 blocks melanoma cells in G2/M phase and induces apoptosis. Cell cycle analysis of nine cell lines treated with 100 nM WP760 (for 24 h) by flow cytometry (**a**). Representative histograms of cell cycle distribution (treated - WP vs. controls - CONTR) are shown. Cell cycle distribution presented as mean ± SD of at least three independent experiments (**b**); * *P*-values <0.05, ** *P*-values <0.01 (Student’s t-test). Western blotting analysis of caspase 3 cleavage in WP760-treated cell lines (**c**). Cells were treated with 100 nM (or 50 nM for 1205Lu) for 24 (*upper*) or 48 h (*lower*); HSP70 (loading control). Figure shows representative results of at least two independent experiments

WP760 induced G2/M-phase cell cycle arrest in 8 out of 9 cell lines. Furthermore, statistically significant inhibition of S phase was observed in WM3248 and 1205Lu cell lines (Fig. 2a, b). WP760 (50 nM and 100 nM) induced apoptosis in most cell lines, as shown by caspase 3 cleavage (Fig. 2c) and TUNEL staining (Supplementary material, Table S4). Depending on cell line, apoptosis was detected as early as 24 or 48 h post-treatment. The only cell line with no apoptosis response to WP760 was WM3928F. Interestingly, caspase 3 cleavage was not observed in 451Lu and WM1382 cell lines; a higher subG1 phase was observed, however, in cell cycle analysis, indicating apoptosis in WM1382 cell line (Fig. 2a).

### WP760 binds to DNA but does not induce double-strand breaks

**Fig. 3:**
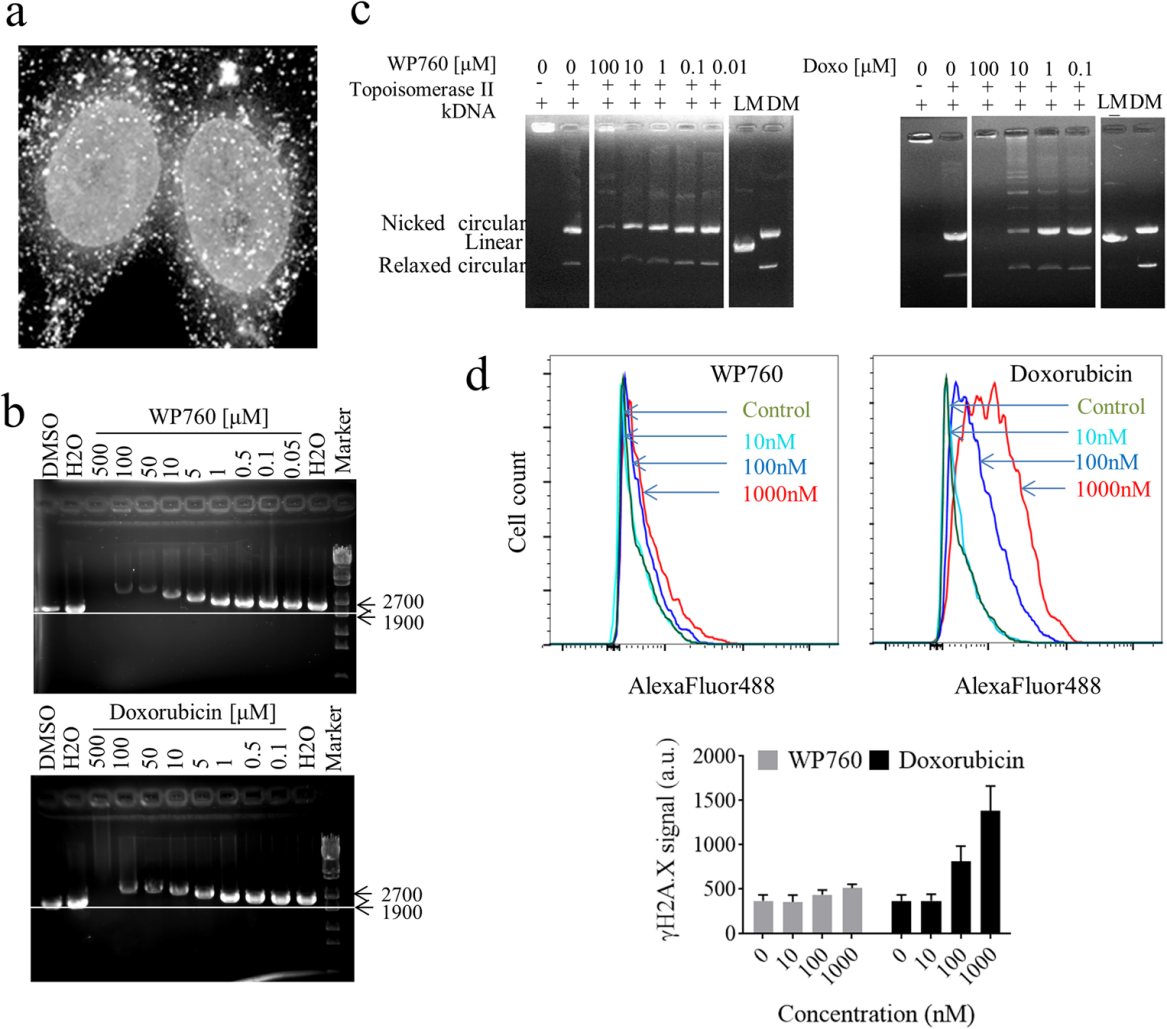
Cellular localization of WP760 and its interaction with DNA. Intracellular localization of WP760 (1 μM) in WM35 cells after 4-h treatment determined by fluorescence microscopy (**a**). Binding of WP760 or doxorubicin to DNA (DNA mobility shift assay), (**b**). Inhibition of topoisomerase IIα by WP760 or doxorubicin (decatenation assay), (**c**). Determination of DNA double strand breaks using histone γ-H2AX immunostaining and flow cytometry (**d**). Representative histograms of γ-H2AX signal intensity in cells treated with various drug concentrations are shown in upper panel. Mean value of γ-H2AX signal ± SD from two separate experiments is shown in lower panel

To examine intracellular drug distribution, WM35 cells were exposed to 1 μM WP760. WP760 intrinsic fluorescence could be seen in cells after 4 h and it was localized predominantly in nuclei (Fig. 3a). A similar time-dependent accumulation pattern was observed for A375 cells exposed to WP760 for 2 and 24 h (Supplementary material, Fig. S1). pEBFP linearized plasmids were incubated with WP760 or doxorubicin (reference) to investigate whether WP760 binds to double-stranded DNA. Concentration-dependent retardation of plasmid migration was found, indicating DNA-drug binding (Fig. 3b).

Binding of anthracyclines to DNA may result in ternary complexes with topoisomerases and inhibited DNA ligation. In vitro decatenation assay was performed to evaluate the effect of WP760 on topoisomerase IIα activity. Poisoning of the enzyme by WP760 prevented formation of decatenation products as shown by agarose gel electrophoresis. Doxorubicin (reference) inhibited topoisomerase II activity at 10 μM, whereas WP760 did so at 100 μM only, indicating its weaker TopoII poisoning capability (Fig. 3c).

We evaluated formation of DNA double-stranded breaks in WP760-treated cells by analyzing (flow cytometry) the intensity of γ-H2AX immunofluorescence signal in WP760-treated WM35 cells (Fig. 3d). The results show no formation of double-strand breaks, even at high WP760 concentration (100-fold higher than cytotoxic IC_50_ value for WM35 cell line). In contrast, doxorubicin treatment significantly increased the γ-H2AX signal. Thus, the mechanism of WP760 cytotoxicity probably does not rely on interference with topoisomerase II catalytic activity.

### WP760 suppresses transcription, induces nucleolar stress, and activates p53

**Fig. 4:**
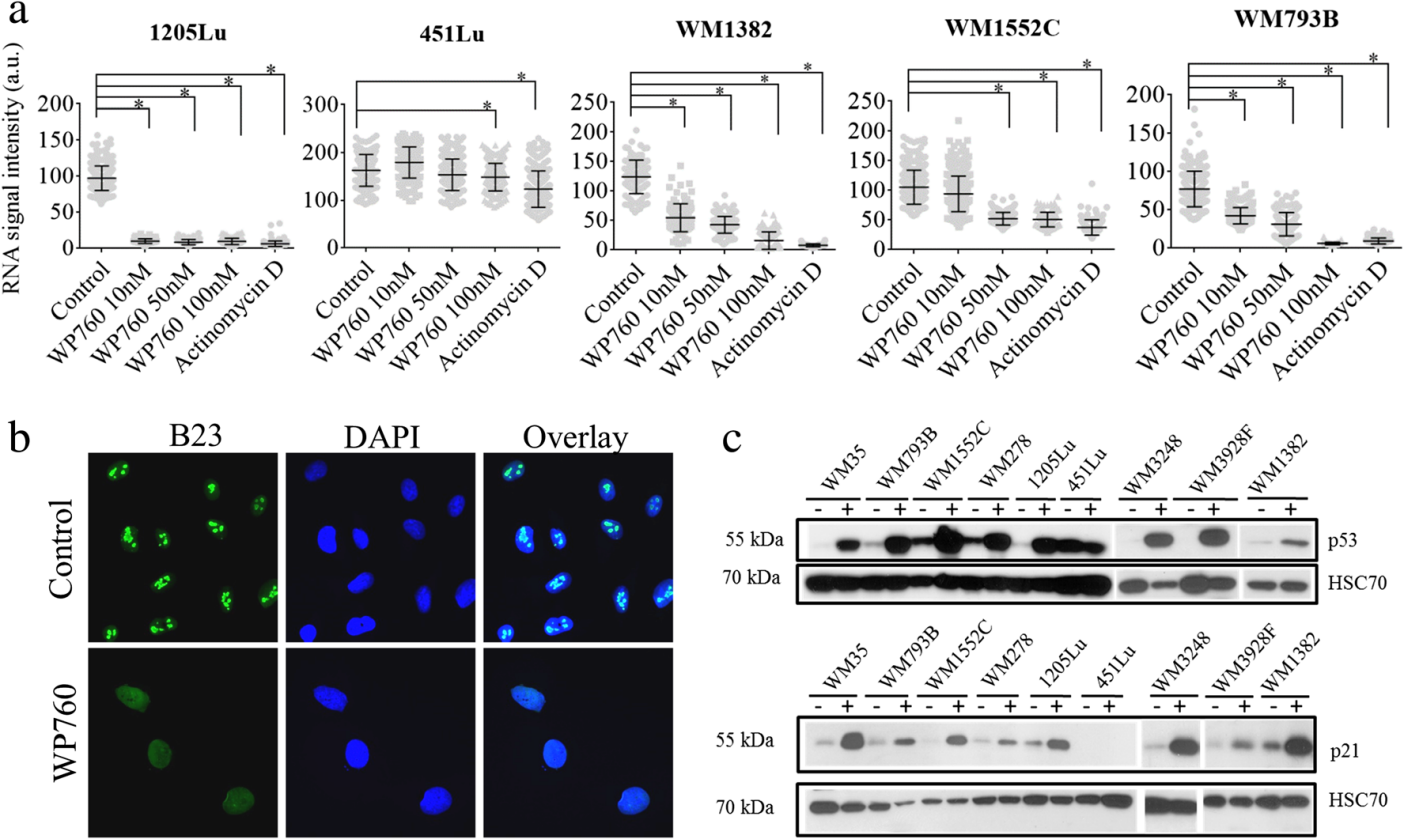
Effect of WP760 and Actinomycin D on global transcription level in melanoma cell lines. Cells were treated with various concentrations of WP760 for 24 h. RNA transcription level was determined by measuring the signal of fluorescently labeled 5-ethynyl uridine (EU) incorporated into nascent RNA. Asterisks indicate statistically significant differences, *P*-values < 0.05 (Fisher test) (**a**). Effect of WP760 on subcellular localization of B23 protein in control and WP760-treated (100 nM; 24 h) WM793B cells (**b**). Western blots of total p53 and p21 in WP760-treated (100 nM; 24 h) lines (**c**). WP760 increases the amount of p53 and p21 significantly and consistently in 8 out of 9 human melanoma cell lines tested; HSC70 (loading control). The panel shows representative results of two independent experiments

Exposure to WP760 resulted in a significant dose-dependent decrease in RNA production in treated cells (Fig. 4a). Actinomycin D (25 nM, reference) completely inhibited the production of new RNA in nearly all tested lines. The least significant difference in the levels of nascent RNA transcription between WP760-treated and control cells was observed for 451Lu cell line. Interestingly, actinomycin D was not as potent in this cell line as in other ones. Because the vast majority (80–90%) of total RNA is ribosomal RNA (rRNA), WP760 should have inhibited mainly the synthesis of rRNA. However, the results of PCR arrays indicate that WP760 affected the synthesis of a broad spectrum of mRNAs (see below).

**Fig. 5:**
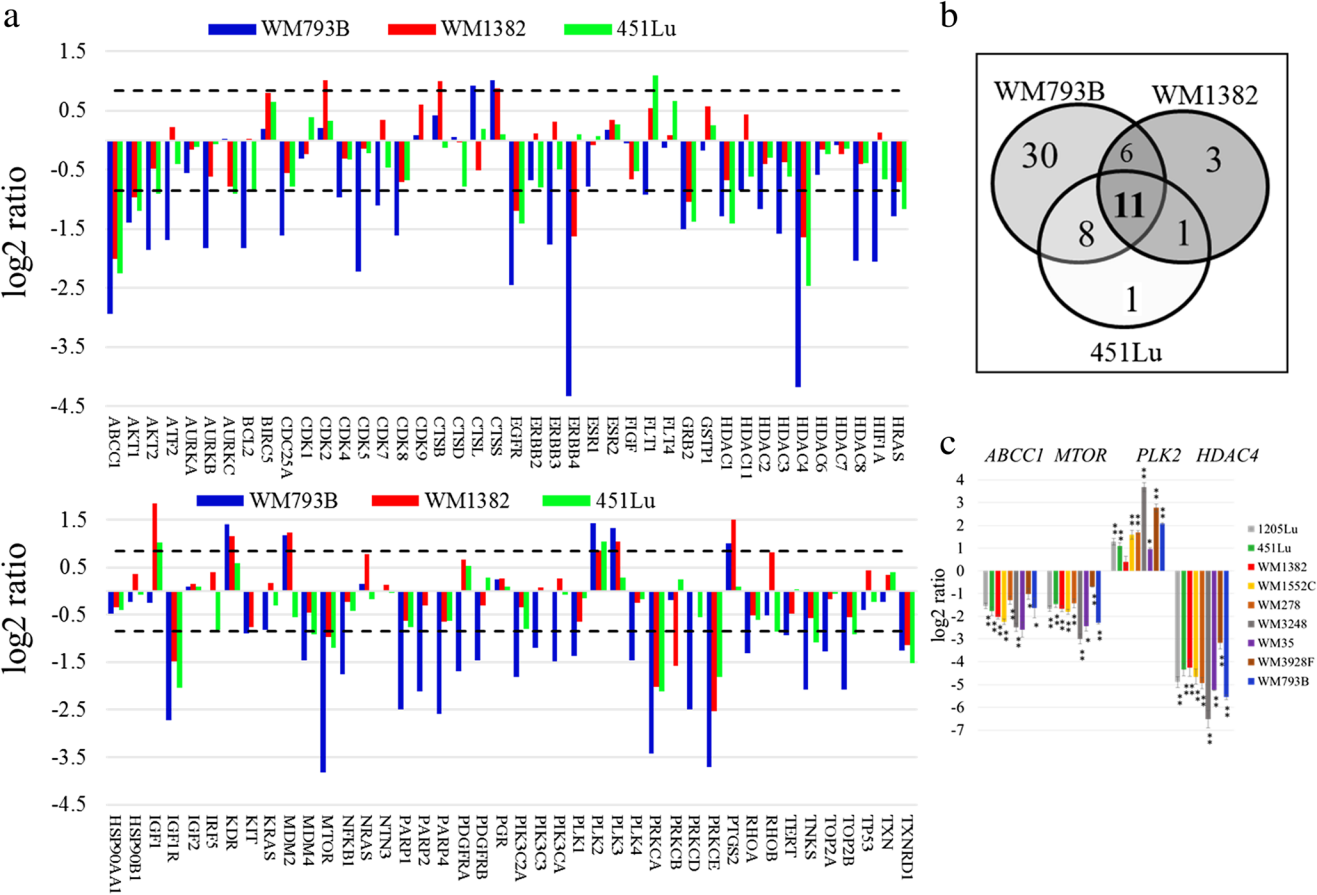
PCR array analysis and validation of results. Gene expression analysis in melanoma cells (WM793B, WM1382 and 451Lu) treated with 100 nM WP760 for 24 h (**a**). The diagram shows log2 ratios for all 84 transcripts (dotted lines signify fold regulation cut-off value of 1.8; Venn diagram showing numbers of genes with altered expression (**b**). qRT-PCR validation of four genes in a panel of 9 cell lines (**c**). The results are presented as mean ± SD of three biological replicas (* *P*-values <0.05, ** *P*-values <0.01; Student’s t-test)

Next, subcellular localization of B23 nucleolar phosphoprotein was evaluated to determine whether WP760 induces nucleolar stress in response to transcription inhibition. B23 is a nuclear chaperone localized in the granular component of nucleolus. Under stress conditions, it translocates to the nucleoplasm [10]. B23 shows predominantly nucleolar localization in control cells, but treatment with WP760 results in diffuse distribution throughout the nucleus (Fig. 4b). Nucleolar integrity, B23 expression, and p53 stability are functionally linked [11–13]. WP760 treatment significantly increases p53 level in all cell lines except 451Lu (Fig. 4c). Activation of the p53 pathway is further corroborated by significant elevation of p21 expression (Fig. 4c). Consistent with increased p53, the level of MDM2 transcript is also increased in two cell lines with wt TP53 (Fig. 5a). In contrast, the level of MDM2 transcript did not change in 451Lu cell line (homozygous TP53 mutant).

### Molecular response of melanoma cells to WP760

We analyzed 84 genes in three melanoma cell lines differing in WP760 sensitivity and genetic background (BRAF and TP53 status) using Human Cancer Drug Targets RT^2^ Profiler™ PCR Array. Approximately 70% confluent cell cultures were treated with WP760 (100 nM/24 h). This treatment significantly altered the expression of 65.5% (55), 25% (21), and 25 (21) of genes (fold regulation cut-off >1.8) in WM793B, WM1382, and 451Lu cell lines, respectively (Fig. 5a,b). Expression of most altered genes was decreased. The highest transcription suppression was observed in WM793B (expression of 57% analyzed genes was reduced by >1.8-fold). Although WP760 treatment caused less profound effect in WM1382 and 451Lu cell lines, transcription inhibition could still be clearly detected.

The suppressed genes with >1.8-fold change in all three analyzed cell lines (WM793B, WM1382, and 451Lu cell lines) regulate drug resistance (*ABCC1*), cell growth (*MTOR, AKT1*), proliferation (*IGF1R, EGFR, GRB2*), protein kinase pathways (*PRKCA, PRKCE*), transcription (*HDAC4*), and other processes (*TXNRD1*). *PLK2*, a tumor suppressor gene, was upregulated in all cell lines. Interestingly, *PLK3* and *MDM2*, both involved in p53 activation, were upregulated in WM793B and WM1382 but not in 451Lu cell line harboring p53 mutation in both alleles.

The expression of four selected genes (*MTOR, ABCC1, PLK2* and *HDAC4*) was validated in nine melanoma cell lines by qPCR. Results of qPCR were similar to the PCR array results (Fig. 5c). Notably, there was a massive *HDAC4* downregulation in all cell lines tested (7–90 fold change).

**Fig. 6:**
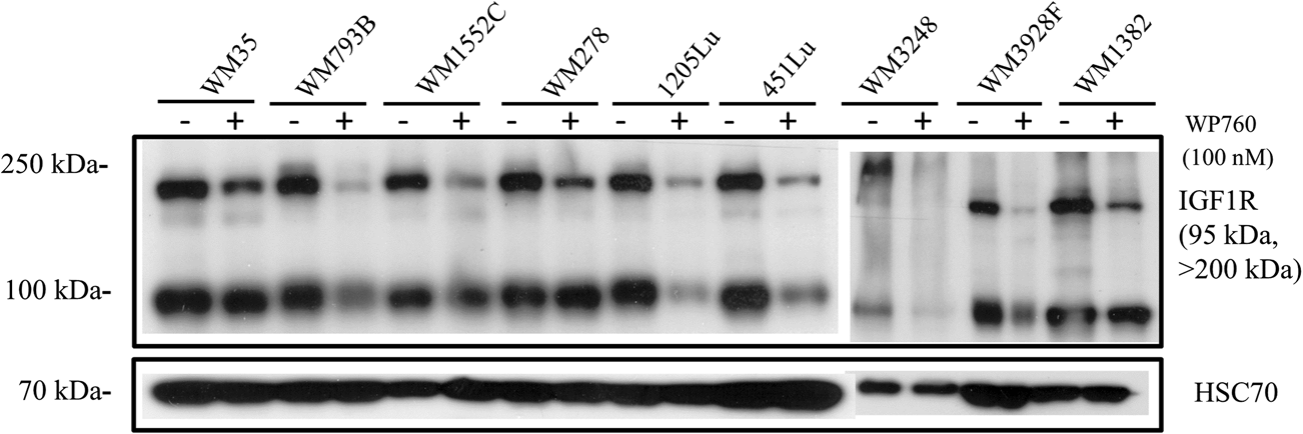
Expression of IGF1R protein in melanoma cells treated with WP760. Western blotting of total IGF1Rβ in WP760-treated (100 nM; 24 h) cells. Samples containing 20 μg of protein were subjected to electrophoresis and immunoblotting. HSP70 was used as a loading control. The figure shows representative results of two independent experiments

Since PCR array results suggested that IGF1R was involved in melanoma cells’ response to WP760, expression of this protein was evaluated. IGF1R was reduced in most of the treated cell lines (Fig. 6); the pre-IGF1 receptor was significantly inhibited in all the tested cell lines, whereas the final form was decreased in 1205Lu, 451Lu, WM3248 and WM3928F cells (2.4–13 fold change). These results suggest that the IGF1R pathway may also be targeted by WP760.

## Discussion

Melanoma is the most aggressive skin cancer with an average survival time of 9 months in advanced disease [14]. The approved targeted drugs can extend life by a few months; immunotherapy shows long-lasting therapeutic effects also only in a minority of patients [15]. Thus, there is an urgent need for novel drugs/strategies that could supplement the current anti-melanoma treatment.

Anthracyclines have not been used in clinical management of melanoma. Nevertheless, WP760, a bis-anthracycline, had demonstrated a high anti-melanoma specificity in NCI-60 cell line antitumor screen [6]. However, relatively few melanoma cell lines were examined in that study and the mechanism of WP760 action was not explored in depth. WP760 showed anti-melanoma activity by activating the p53 pathway, suppressing the MAPK pathway and iNOS [6]. We investigated in here the mechanism of WP760 action in melanoma although our results suggested that WP760 may exert inhibitory effect also in other types of cancer cells.

We evaluated inhibitory activity of WP760 for well-established molecular targets of anthracyclines such as DNA damage and RNA synthesis inhibition. Interaction of WP760 with DNA was examined to assess its capability of inducing DNA damage via topoisomerase inhibition, which has been known as one of primary mechanisms of doxorubicin and daunomycin antitumor activity [3]. Although WP760 inhibited topoisomerase II in a cell-free assay at high concentrations (exceeding IC_50_ by several dozen folds), it was not able to induce DNA damage in cells. These findings suggest that the prevailing mechanism of WP760 cytotoxicity does not rely on inhibition of topoisomerase II.

Since binding of drugs to DNA may affect RNA synthesis, the level of nascent RNA was evaluated in WP760-treated cells. A dramatic decrease in newly synthesized RNA levels was observed. Global inhibition of RNA synthesis results predominantly from blocked synthesis of rRNA, but in our study a decrease of some mRNA transcripts could also be observed. PCR array analysis demonstrated that WP760 significantly inhibited expression of 20–57% of the analyzed genes.

Transcription inhibition is generally regarded as a potent cellular stress-inducing factor. WP760-induced stress was strongly indicated by changes in nucleolus structure (B23 relocation from nucleolus to nucleoplasm), which was observed in most examined cell lines. It is well-established that stress-inducing stimuli disrupt nucleolar integrity and cause B23 relocation, leading to p53 activation [12, 13] and growth arrest. Moreover, nucleoplasmic B23 enhances p53 stability by sequestration of MDM2 [16], a well-known p53 regulator. In the present study, activation of the p53 pathway was indicated by substantially increased expression of p21 protein in almost all of the studied cell lines, as well as by increased MDM2 mRNA expression, as shown by the PCR array study (in two cell lines). Although this mode of action was described for several drugs including doxorubicin [17], our data indicate that doxorubicin is not as potent as WP760 in inhibiting melanoma cell growth. This suggests a more complex mode of WP760 action. Anthracyclines can induce mutant p53 accumulation by increase of p53-encoding mRNA [18]. This could explain huge induction of p53 in WM1552C cells. Other possible targets are: TopoI inhibition [19], direct release of cytochrome c from mitochondria [20] or inhibition of AKT and protein synthesis [21].

In search for molecular targets of WP760 we performed PCR array analyses. They demonstrated expression of *HDAC4*, *MTOR*, *PLK2*, and *ABCC1* to be affected in all melanoma cell lines tested. Additionally, decreased expression of pre-IGF1R or IGF1R was observed, suggesting that WP760-mediated IGF1R inhibition may contribute to its anti-melanoma activity. The IGF1R pathway was reported to be involved in melanocyte transformation [22], aggressiveness and stemness [23, 24] as well as therapy resistance [25]. Targeting of the IGF1R pathway exerts potent anticancer effects [26, 27] and combined inhibition of MAPK, PI3K, and IGF1R successfully inhibited melanoma growth and overcame resistance of melanoma cells harboring BRAF and PTEN mutations [28]. Here, we also observed that cell lines, both BRAF- and PTEN-mutated, were the most sensitive towards WP760, however further studies are required to prove that inhibition of IGF1R indeed contributes to WP760 anti-melanoma activity. Treatment with WP760 resulted also in huge suppression of *HDAC4* in all cell lines (several dozen times). Histone deacetylases are attractive therapeutic targets in melanoma [29, 30], especially in combination with immunotherapy and targeted therapy [31], thus further validation of this mechanism of WP760 action is strongly warranted. It would be interesting to investigate whether this inhibition is specific or just results from global RNA synthesis inhibition. We also confirmed WP760 ability to activate p53. TP53 is mutated in a relatively small population of melanoma cases (17%); in most melanomas nonetheless this pathway is inactivated due to *MDM2* or *MDM4* amplification, *CDKN2A* deletion, or deregulation of ASPP. Still, in approximately 85% of melanoma patients structurally wild-type p53 is expressed and reactivation of this pathway is possible, as suggested by in vitro restoration of p53 [32, 33]. The rationale for p53 activation in melanoma is further strengthened by recent data suggesting that activation/reactivation of p53 sensitizes melanoma cells to vemurafenib, irrespective of TP53 status [34]. WP760 is a strong activator of p53. Out of the nine melanoma cell lines tested in this study, only one (451Lu harboring homozygous TP53 mutation) did not respond to WP760 by p53 activation. Nevertheless, proliferation of this cell line was significantly inhibited by WP760, suggesting that p53-activated apoptosis is not the unique mechanism of WP760 action.

In conclusion, the present study identified WP760 as a novel transcriptional inhibitor with potent ability to activate p53 in melanoma cells. These properties and the ability to inhibit IGF1R and *HDAC4* make WP760 an attractive potential chemotherapeutic candidate worthy of further investigation.

## Electronic supplementary material

ESM 1 (PDF 213 kb)
