# Supplementary material for: Bis-anthracycline WP760 abrogates melanoma cell growth by transcription inhibition, p53 activation and IGF1R downregulation
Source: Invest New Drugs. 2017 Apr 17;35(5):545–55. doi: 10.1007/s10637-017-0465-9 (PMC5613070; doi:10.1007/s10637-017-0465-9)
Supplement: Supplementary file 1 — (PDF 213 kb) [file 10637_2017_465_MOESM1_ESM.pdf]

Bis-anthracycline WP760 abrogates melanoma cell growth by transcription inhibition and p53 activation

Magdalena Olbryt[1], Aleksandra Rusin, Izabela Fokt, Anna Habryka, Patrycja Tudrej, Sebastian Student, Aleksander Sochanik, Rafał Zieliński, Waldemar Priebe

[1]Center for Translational Research and Molecular Biology of Cancer, Maria Skłodowska-Curie Memorial Cancer Center and Institute of Oncology, Gliwice Branch, Gliwice, Poland

Investigational New Drugs

Supplementary material

Table S1. Cell lines

Name	Medium	Repositorium
1205Lu, 451Lu, WM1552C, WM278, WM35	Tu2%	ATCC
WM1382, WM3248, WM3928F	Tu2%	Coriell Cell
A375, Mel1617, SB2, SKMel-28, WM793B VR, SKMel-28 VR, Mel1617 VR, A375 VR	RPMI1640 5% FBS	MD Anderson*
A549	DMEM 10% FBS	ATCC
	RPMI 10% FBS	derived by us from ovarian cancer patient's ascites fluid

* Cells were kindly provided by Professor Menashe Bar-Eli, Department of Cancer Biology, UT M.D. Anderson Cancer Center, Houston, Texas, USA

Table S2. Sequences of the primers used in qRT-PCR

Gene	(National Center for Biotechnology Information reference sequence number)	Left primer	Right primer
ABCC1	XM_011522498.1<br>XM_011522497.1<br>NM_004996.3	ccatgtgggaaaacacatctt	ctgtgcgtgaccaagatcc
HDAC4	NM_006037<br>NP_006028	ccatggagaaagtcatggaga	tctgagcctcgatcagagaac
MTOR	XM_005263438.1<br>NM_004958.3	tgctggaagccctttgtctatg	cgcttgttgcctttggtatt
PLK2	NM_006622.3	agatctcgcggattatcgtc	tgtcaaatctgtcatctcgtaaca
RPLPO	NM_001002.3<br>NM_053275.3	gatgcccagggaagacag	acaatgaaacatttcggataatca
B2M	NM_004048.2	ttctggcctggaggctatc	tcaggaaatttgactttccattc
PLXNB2	NM_012401.2	ggccgtcatctgcaactc	taaggaggagctggatggtc

Table S3. Antibodies used in Western blotting

Antibody	Dilution	Firm	Cat. No.
anti- cleaved Caspase 3 (Asp175) (5A1E)	1:2000	Cell Signaling Technology	#9664
anti-HSC70 (B-6)	1:3000	Santa Cruz Biotechnology	SC-7298
anti-IGF1Rbeta (D23H3)	1:2000	Cell Signaling Technology	#9750
anti-P21 (F-5)	1:500	Santa Cruz Biotechnology	SC-6246
anti-TP53 (DO-1)	1:2000	Santa Cruz Biotechnology	SC-126
Goat anti-Rabbit IgG (H+L), HRP	1:2000	Thermo Fisher Scientific Pierce	#31460
Goat anti-Mouse IgG (H+L), HRP	1:2000	Thermo Fisher Scientific Pierce	#31430

Table S4. Apoptotic index of melanoma cells treated with 100 nM WP760 assessed with TUNEL assay.

	**Apoptotic index (%)**	
**Cell line**	**Control**	**Treated**
	**24h**	
**WM1552C**	0.4	2.5
**WM278**	0.9	2.0
**WM3248**	1.0	6.4
**WM35**	1.8	7.5
**WM793B**	1.2	6.4
	**48h**	
**1205Lu**	0.8	9.9*
**451Lu**	0.6	4.0
**WM1382**	0.4	4.5
**WM3928F**	0	0

*WP760 – 50 nM

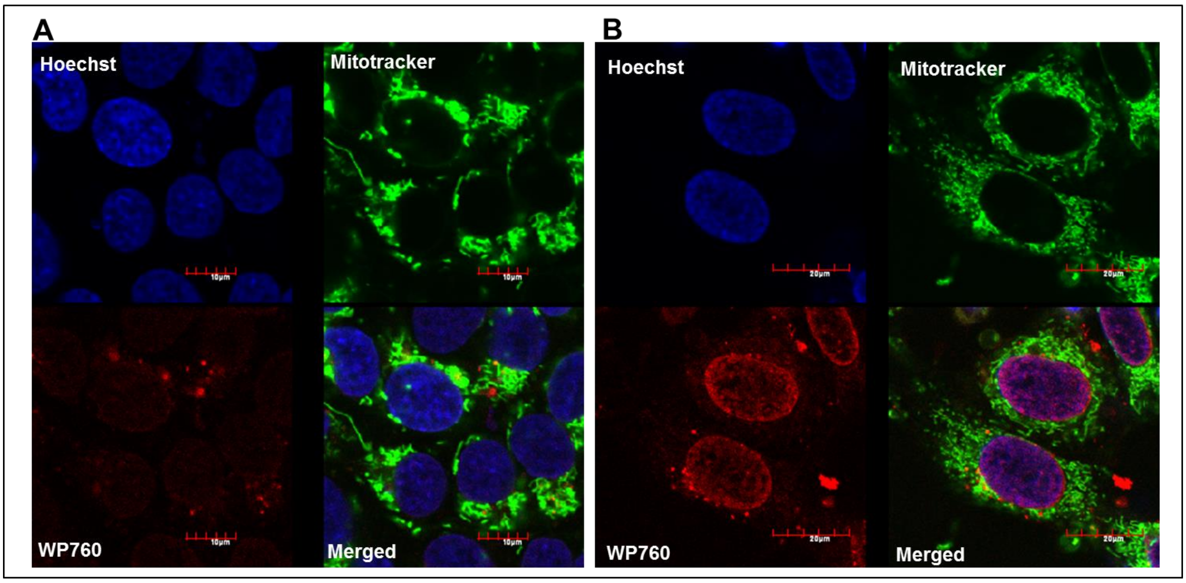

Fig. S1. Accumulation of WP760 in the nucleus of A-375 cells. A-375 cells plated on chambered coverglass were exposed to WP760 (1 µM) for 2 (A) and 24 h (B) followed by imaging using Olympus FLV1000 confocal system. Hoechst 33342 (blue) at 1 µg/ml and 75 nM Mitotracker (green) was used for nuclei and mitochondria counter staining
